# Supplementary material for: A Comprehensive Proteomic SWATH-MS Workflow for Profiling Blood Extracellular Vesicles: A New Avenue for Glioma Tumour Surveillance
Source: Int J Mol Sci. 2020 Jul 3;21(13):4754. doi: 10.3390/ijms21134754 (PMC7369771; doi:10.3390/ijms21134754)
Supplement: Supplementary file 1 [file ijms-21-04754-s001.zip › Supplementary Figures S1-S7.docx]

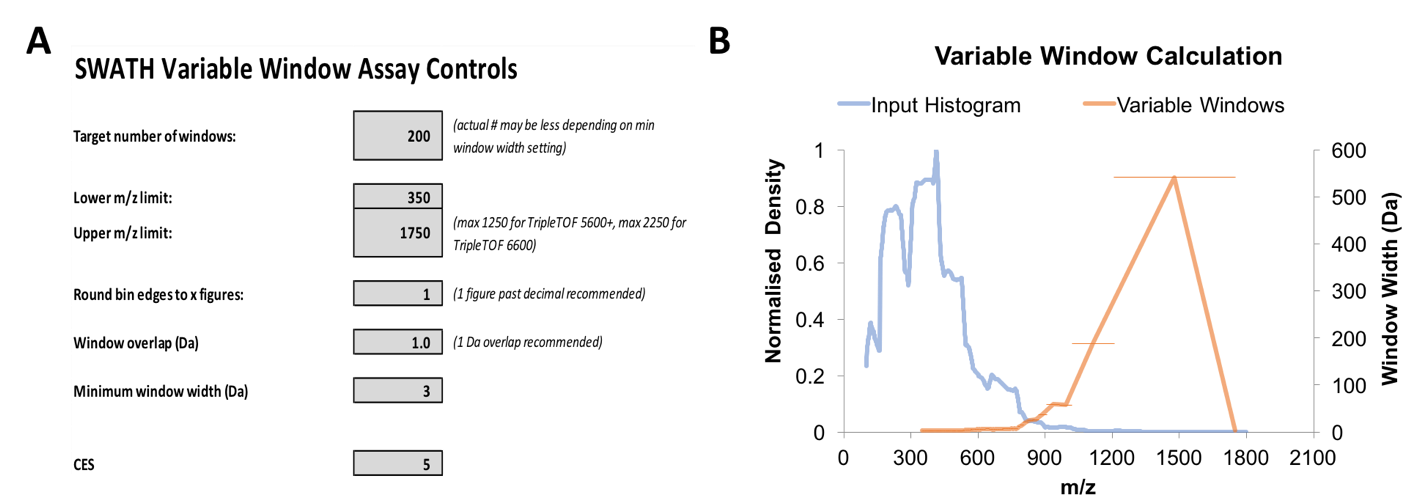


**Figure S1**. **SWATH assay parameters** (A) Parameters used to generate SWATH variable windows. (B) Graph of the *m/z* histogram and the variable swath windows that were generated. Smaller *m/z* window ranges were generated for higher inputs.


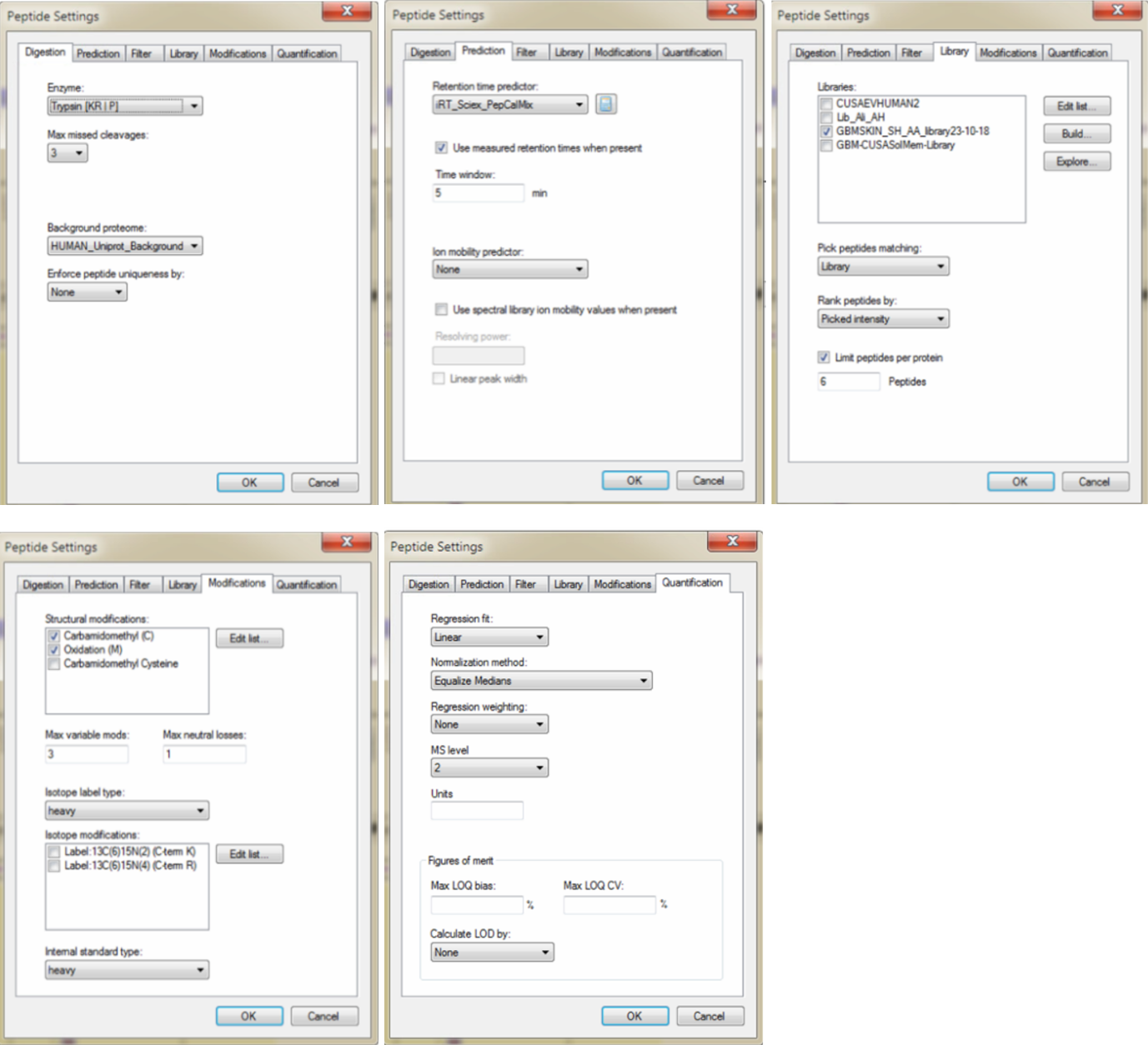


Figure S2. Peptide settings used for the analysis of SWATH-MS data in Skyline4.2.


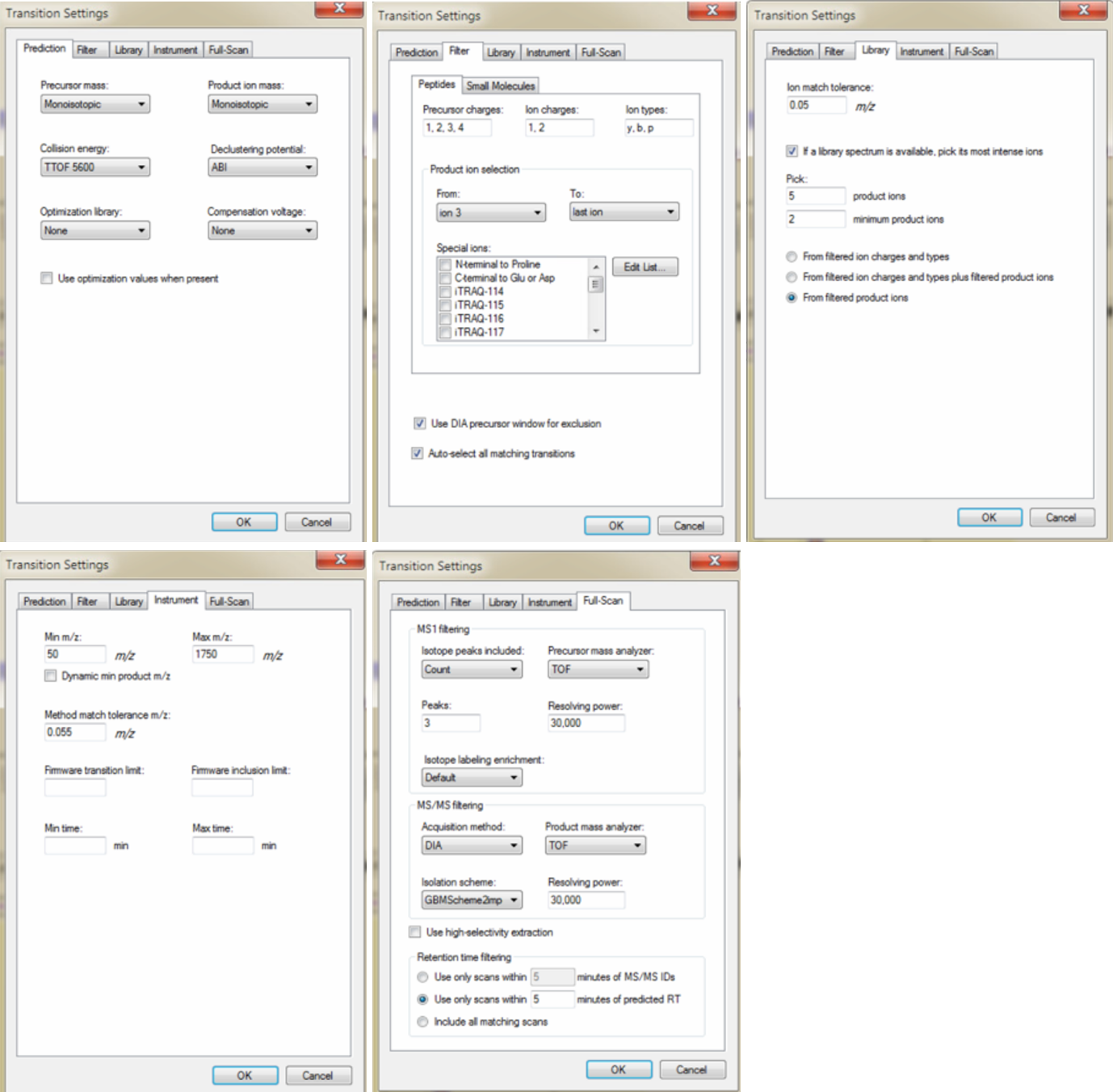


**Figure S3. Transition settings used for the analysis of SWATH data in Skyline4.2**.


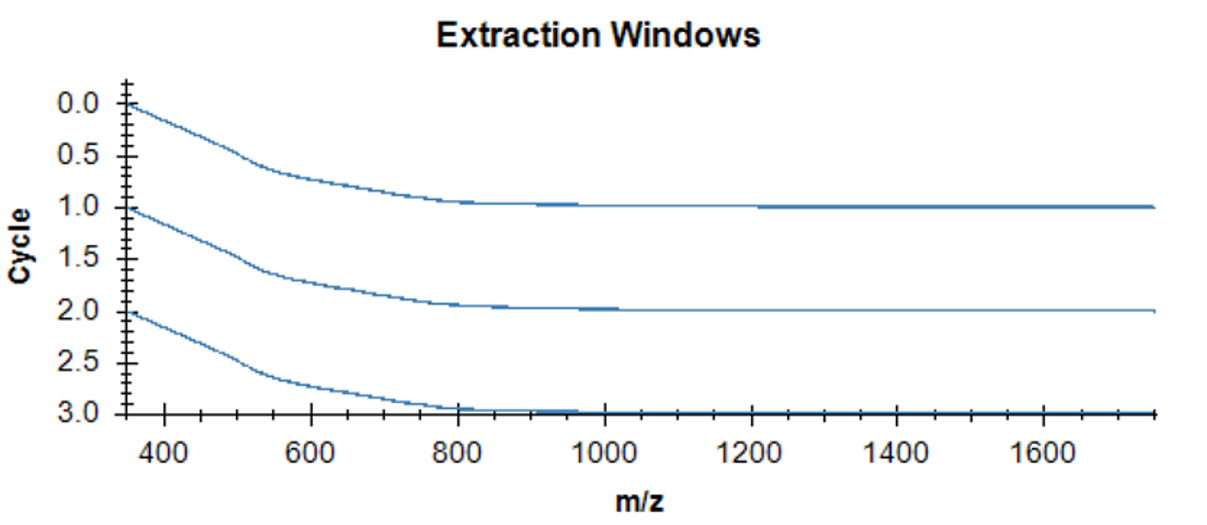


**Figure S4.** Skyline4.2 isolation scheme of the variable isolation windows used on the TripleTOF®6600 to acquire SWATH-MS data.

**
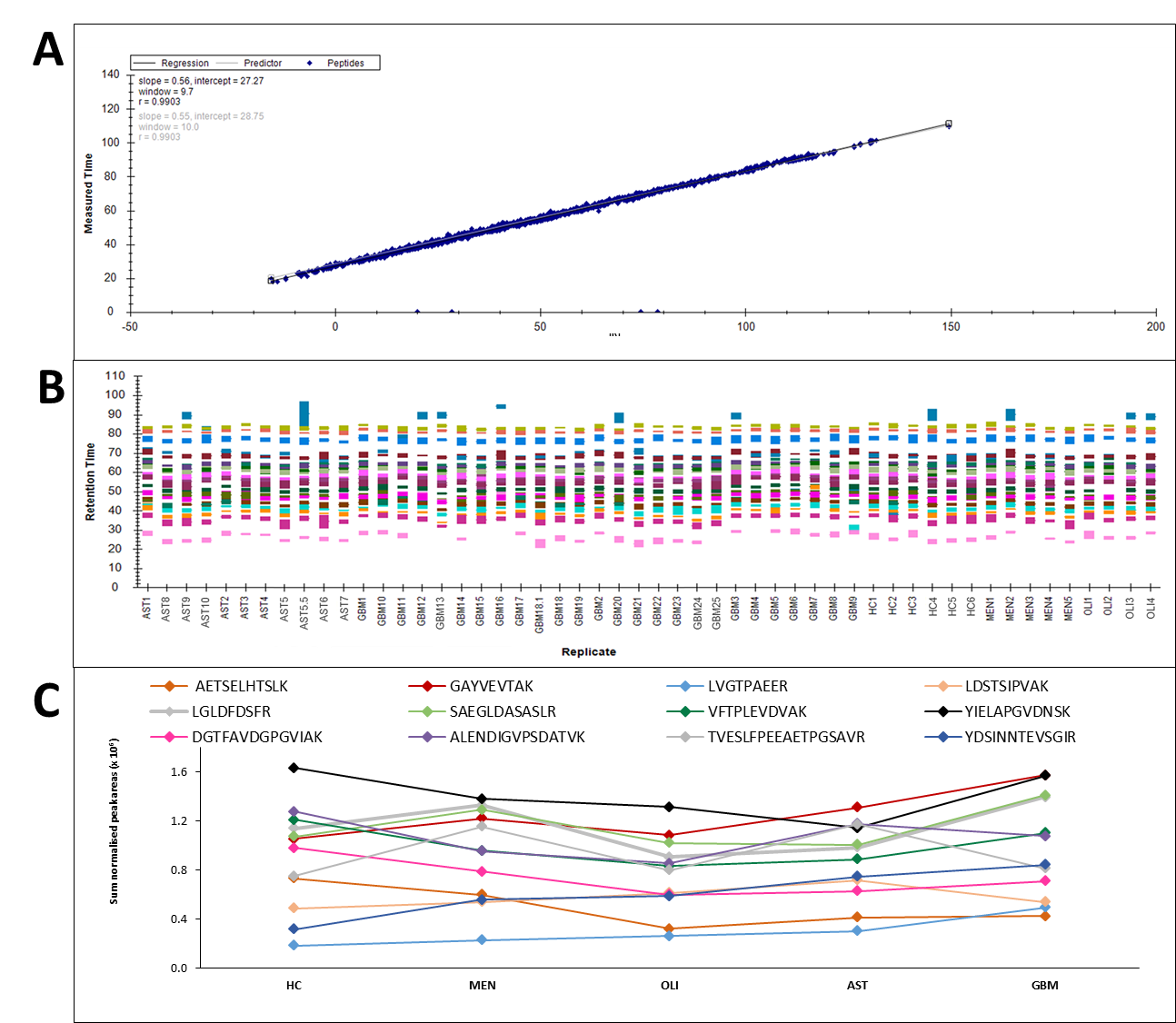
**

**Figure S5. Data quality and reproducibility for glioma plasma-EVs**. (A) iRT calculator for the 20 *PepCalMix* peptides to allow retention time prediction and alignment of the SWATH-MS files to the spectral library. The *PepCalMix* peptides spiked-in to the spectral library were calibrated to the *PepCalMix* iRT definition values in Skyline4.2. The iRT linear regression between the measured retention times of the peptides (y-axis) versus their respective iRT definition values (x-axis) had a Pearson’s correlation coefficient (*r*) of 0.9903. (B) The calibrated retention time ranges for 20 *PepCalMix* peptides for plasma EV samples analysed by SWATH-MS. (C) Scatter plot of the total peak area assigned to 12 PepCalMix peptides for patient plasma EVs categorised by genetic-histological subtype.

**
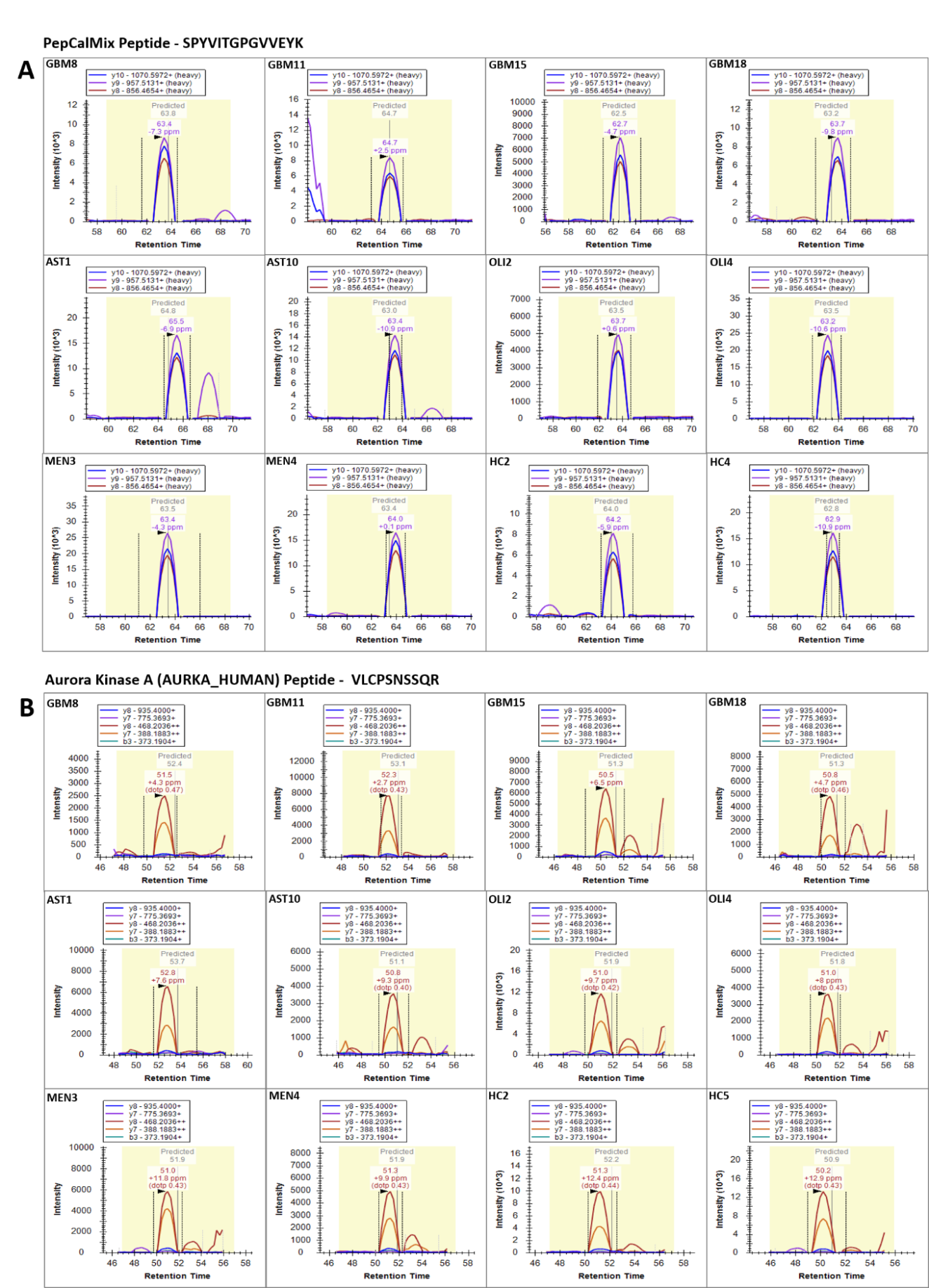
**

**Figure S6. Extracted ion chromatograms (XIC) of standard PepCalMix and Aurora Kinase A peptide.** (A) XIC for the PepCalMix peptide SPYVITGPGVVEYK, and its product ions y8 (856.4654+; red), y9 (957.5131+; purple) and y10 (1070.5972+; red) across 12 plasma EV samples analysed by SWATH-MS. (B) The XIC for Aurora Kinase A (AURKA_HUMAN) peptide VLCPSNSSQR (574.2799 +++ (dotp 0.38)) and its product ions y8 (935.4000+; blue), y7 (7875.3693+; purple), y8 (468.2036++; red), y7 (388.1883++; orange) and b3 (373.1904+; green) across the same 12 samples.

Figure S7. Bioinformatics of significant proteins in GBM plasma-EVs relative to healthy controls (⎪FC⎪≥2, adj. *p*≤0.05). (A) PANTHER over-representation test results for biological processes and reactome pathways; over/under-represented proteins relative to the number of proteins expected in the PANTHER category. (B) Ingenuity functional pathway analysis shows top canonical pathways, diseases and disorders, and molecular and cellular functions associated with the significant protein changes (listed with the numbers of overlapping molecules and significance of associations; right-tailed Fisher exact test, *p* value.
